# Sustaining nitrogen dynamics: A critical aspect for improving salt tolerance in plants

**DOI:** 10.3389/fpls.2023.1087946

**Published:** 2023-02-23

**Authors:** Faroza Nazir, Moksh Mahajan, Sayeda Khatoon, Mohammed Albaqami, Farha Ashfaque, Himanshu Chhillar, Priyanka Chopra, M. Iqbal R. Khan

**Affiliations:** ^1^ Department of Botany, Jamia Hamdard, New Delhi, India; ^2^ Department of Botany and Microbiology, College of Science, King Saud University, Riyadh, Saudi Arabia; ^3^ Department of Botany, Aligarh Muslim University, Aligarh, India

**Keywords:** crop productivity, genetic engineering, nitrogen metabolism, phytohormones, salt stress resilience

## Abstract

In the current changing environment, salt stress has become a major concern for plant growth and food production worldwide. Understanding the mechanisms of how plants function in saline environments is critical for initiating efforts to mitigate the detrimental effects of salt stress. Agricultural productivity is linked to nutrient availability, and it is expected that the judicious metabolism of mineral nutrients has a positive impact on alleviating salt-induced losses in crop plants. Nitrogen (N) is a macronutrient that contributes significantly to sustainable agriculture by maintaining productivity and plant growth in both optimal and stressful environments. Significant progress has been made in comprehending the fundamental physiological and molecular mechanisms associated with N-mediated plant responses to salt stress. This review provided an (a) overview of N-sensing, transportation, and assimilation in plants; (b) assess the salt stress-mediated regulation of N dynamics and nitrogen use- efficiency; (c) critically appraise the role of N in plants exposed to salt stress. Furthermore, the existing but less explored crosstalk between N and phytohormones has been discussed that may be utilized to gain a better understanding of plant adaptive responses to salt stress. In addition, the shade of a small beam of light on the manipulation of N dynamics through genetic engineering with an aim of developing salt-tolerant plants is also highlighted.

## Introduction

**Figure 1:**
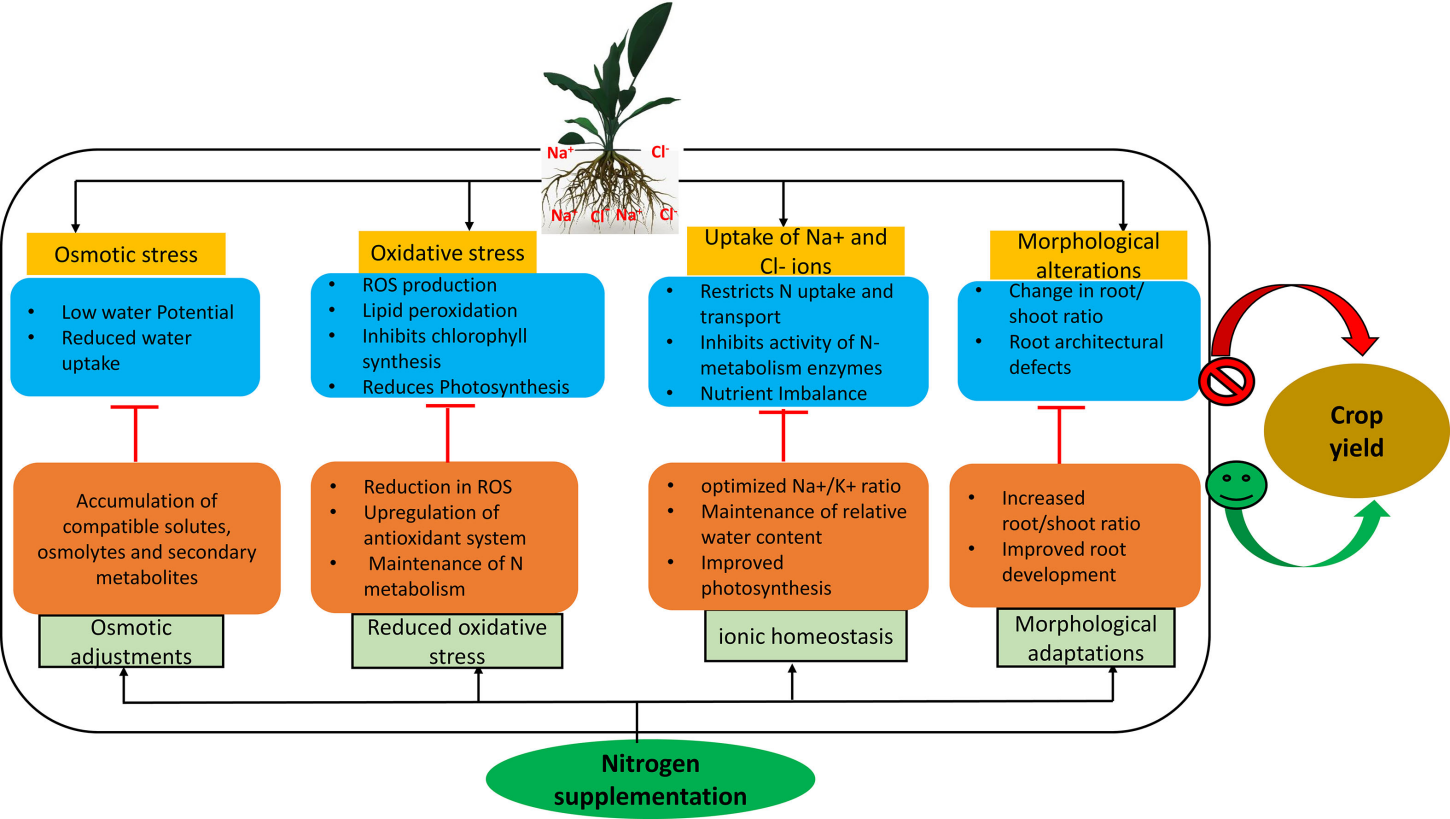
N supplementation exerts a defensive response on plant physiological and morphological machinery during salt stress and helps in alleviating the osmotic stress, oxidative damage and morphological alterations. N also help in maintaining the ionic homeostasis along with N metabolism in plants during salt stress, which in turn helps in improving the plant performance during salt stress. Cl^-^, chloride; N, Nitrogen; Na^+^, sodium; K^+^, Potassium; ROS, reactive oxygen species.

Soil salinization is a detrimental ecological issue, limiting crop production, quality and global problem for more than half of all arable lands by 2050 (Qureshi and Daba, 2020; Naz et al., 2022). Due to the increased saline levels in the agricultural soils, nitrogen (N) status of soil depleted (Prasad et al., 1997), and it is predicted that up to 50% fertile land will be lost by the middle of the 21st century (Aslam et al., 2017). Mineral nutrient research provides an operative way for exploring plant acclimation to salt stress and improving plant growth and productivity (Torres Bazurto et al., 2017; Sousa et al., 2021). Nitrogen is an indispensable potent nutrient that mediates defense responses, along with a wide range of cellular, physiological and molecular responses critical to plant survival, and various signal transduction pathways associated with plant defense mechanisms and salt-stress resilience (Krapp, 2015; Zhang et al., 2020a; Zhang et al., 2020b). Research focus on sustaining N dynamics in managing salt stress responses gained attention. Salt stress has an impact on nitrification and ammonification in the soil as chloride (Cl^-^) competes with nitrate (NO_3_-) and causes ion toxicities and ionic disparities, which can explicitly restrict N uptake, transport and assimilation processes (Campbell, 1999; Zhao et al., 2020; Liu et al., 2022; Tzortzakis et al., 2022, **Figure 1**). Reductions in N uptake and metabolism have been reported in many plant species (Dalio et al., 2013; Singh et al., 2016; Shahzad et al., 2017; Fuertes-Mendizábal et al., 2020, **Table 1**). For instance, salt stress inhibited NO_3_^-^ and ammonium (NH_4_^+^) uptake in *Zea mays* (maize) leaves (Fuertes-Mendizábal et al., 2020), and may decrease the proportion of N-transport amino acids: asparagine (Asn), glutamate (Glu), aspartate (Asp) and glutamine (Gln) in *Cajanus cajan* (pigeon pea; Dalio et al., 2013) and *Oryza sativa* (rice; Shahzad et al., 2017). Further, salt stress reduced water uptake, N content and nitrate reductase (NR) activity in *Triticum aestivum* (wheat; Ashfaque et al., 2014), inhibited activities of N-assimilation enzymes such as NR, nitrite reductase (NiR), glutamine synthetase (GS) and glutamate oxyglutarate aminotransferase cycle (GOGAT) in *Cucumis sativus* (cucumber) and *Lycopersicon esculentum* (tomato) seedlings (Shao et al., 2015; Singh et al., 2016, **Figure 1**). However, Meng et al. (2016) reported that salt stress has only minimal effect on NR and NiR activity while the substrate concentration (NO_3_^-^ supply) impact NR and NiR activity in *Populus simonii* (Chinese poplar), GS and GOGAT activity were significantly lower in salt-treated plants in comparision to control plants. They presume that this effect is due to decrease in NH_4_^+^ uptake *via* roots under salt stress. Salt stress also restricts NO_3_- uptake on the tonoplast membrane and causes an increase in NH_4_^+^ accumulation in shoots, disrupting N metabolism, and finally yield in rice seedlings (Thu Hoai et al., 2003, **Figure 1**). Thus, the detrimental effects of salt stress on plant metabolism could be attributed to changes in N uptake as well as the activities of various enzymes and genes involved in N metabolism (Chakraborty et al., 2018; Isayenkov and Maathuis, 2019).

**Table 1:** Effect of salt stress on N metabolism and NUE in different plants.

Plants	Salt concentration	Gene expression	Action	References
Rice *(Oryza sativa)*	0.3% NaCl	*OsNRT1.1*, *OsNRT1.5*, *OsNRT1.8*, *OsNRT2.1*, *OsAMT1.1*, *OsAMT1.2*, *OsNR1*, *OsGS1.2*, *OsNADH-GOGAT*, *OsFd-GOGAT*, *OsAS*	Inhibition of NO_3_¯content, NR, GS and NADH-GOGAT activities however, NH_4_^+^ content, Fd-GOGAT, GDH and AspAT activities ↑, decreased expression of *NR1*	Decui et al., 2020
Maize *(Zea mays)*	100 and 430 mM NaCl	*GS1* and *GS2*	Reduced NO_3_¯ content, N assimilation, free amino acids and soluble protein	Fuertes-Mendizábal et al., 2020
Rice	100 mM NaCl	*AMT1.1*, *AMT1.2*, *NIA1*, *NIA2*	N content and accumulation ↓, NR and GS activities ↓, while, NH_4_^+^ uptake ↑	Huang et al., 2020
Tomato (*Solanum lycopersicum* and *Solanum pimpinellifoilum*)	75 mM NaCl	*SlNR*, *SlNiR*, *SlAMT1*, *AlAMT2*, *SlNRT1.2*, *SlGDH*, *SlGS*	N-NO_3_¯, NR, NiR, GS, GDH activities and amino acid accumulation ↓, while, GOGAT activity ↑	Lopez-Delacalle et al., 2020
Beet root *(Beta vulgaris)*	NaCl and Na_2_SO_4_ (2:1M)	*ALN*, *XDH*	Down-regulation of *ALN* while, allantoin, betaine, L-citrulline and melatonin content ↑ and altered N metabolism	Liu et al., 2020
Common bean (*Phaseolous vulgaris*)	100 mM NaCl	*NLP7*, *DREB2A*	*NLP7*, NO_3_¯, NiR and GOGAT activity ↓, however, NH_4_^+^ content and NR activity ↑	Ozfidan-Konakci et al., 2020
Peanut (*Arachis hypogaea*)	200 mM NaCl	NO_3_- transporter coding genes	Expression of NO_3_¯ transporter coding genes repressed thus, reduced N metabolism	Zhang et al., 2020b
Cucumber (*Cucumis sativus*)	80 mM NaCl	*CsPIP1-2*, *CsPIP2-4*	NO_3_¯ uptake, NR, GS, GOGAT, AspAT, AlaAT and IDH activity ↓, conversely NH_4_^+^ uptake and GDH activity ↑	Li et al., 2019
Cucumber	25-75 mM NaCl	*NR*, *NiR*, *GS*, *GOGAT*, *GPT*, *GDH*	NO_3_¯ content, NR, NiR, and GOGAT activities ↓, while, GS and GDH activity ↑	Ma et al., 2019
Rice	100, 200 and 300 mM NaCl	*NR1, NR2, NR3*	Reduced NR activity in salt sensitive genotype	Rohilla and Yadav, 2019
Common grass (*Zostera marina*)	0.4 mM NaCl	*NR*, *NiR*, *NAR*, *NRT2.1*	Up-regulation of NR and NiR gene, and increased NO_3_¯ assimilation	Lv et al., 2018
Tomato (*Solanum lycopersicon *and *Solanum pennelli*)	100 mM NaCl	*NRT1.1*, *NRT1.2*, *AMT1.1*, *AMT1.2*, *GS1*, *NR*	NO_3_¯content ↓, NH_4_^+^ content ↑, down-regulation of *NRT1.1, NRT1.2, AMT1.2, GS1* and NR, while, up-regulation of *AMT1.1*	Abouelsaad et al., 2016
Chinese poplar (*Populus simonii*)	75 mM NaCl	*AMT1.2*, *AMT1.6*, *NRT1.1*, *NRT2.4a*, *NR*, *NiR*, *GS1.3*, *GS2*, *Fd-GOGAT*, *NADH-GOGAT*	NH_4_^+^ uptake ↑, NO_3_¯ uptake and NH_4_^+^ assimilation ↓, weakened GS/GOGAT pathway	Meng et al., 2016
Mustard (*Brassica juncea*)	150 mM NaCl	*NRT1.1*, *NRT1.5*, *NRT2.1*, *AMT1.2*, *AMT2*, *GS1.1*, *GDH1*, *ASN2*, *NiR1*, *GDH2*	Reduced NO_3_^-^ uptake and mobilization, down-regulation of N assimilation associated genes	Goel and Singh, 2015
Cucumber	84 mM NaCl	*NR*, *GS*, *GOGAT*, *β-actin*	NR, GS, GOGAT, GDH activity and soluble protein content↓, proline content↑	Shao et al., 2015
Arabidopsis *(Arabidopsis thaliana)*	100 mM NaCl	*NPF2.3*, *NPF7.3*	Reduced NO_3_¯ translocation from root to shoot	Taochy et al., 2015
Sweet potato *(Ipomoea batatas)*	100 mM NaCl	*NR2*, *NiR2*, *GS2*, *NADH-GOGAT*, *NRT1.1*, *CLCc*	NO_3_^-^ net influx↑, NH_4_^+^ assimilation ↑	Yu et al., 2016
Barley *(Hordeum vulgare)*	150 mM NaCl	*P5CS*	*P5CS* activity and Ala accumulation↑, N metabolism↑	Averina et al., 2014
Maize	25, 75 and 150 mM NaCl	*ZmPAL1*	GS and GOGAT↓, proline accumulation↑, up-regulation of *PAL1*	Ertani et al., 2013
Rice	100 mM NaCl	*OsGS1*, *OsGS2*, *OsNR1*, *OsFd-GOGAT*	Down-regulation of NH_4_^+^ assimilation genes, NO_3_^-^ deficiency	Wang et al., 2012a
Soybean *(Glycine max)*	50, 100 and 200 mM NaCl	*GS*, *GOGAT, HO-1*	Decreased leghemoglobin content, nitrogenase and GS/GOGAT activity while, NH_4_^+^ content ↑	Zilli et al., 2008
Tex-Mex tobacco *(Nicotiana plumbaginifoia)*	300 mM NaCl	*gdhA, gdhB*	Increased NADH-GDH activity and *gdhA* level while, *gdhB* ↓	Restivo, 2004

↑: increased; ↓: decreased; ALN, Allantoinase; XDH, Xanthine dehydrogenase; IDH, Iso-citrate dehydrogenase; PAL, Phenyl ammonia lyase; P5CS, Pyrroline-5-carboxylate synthase; PIP, Plasma membrane intrinsic protein; DREB, Dehydration-responsive element-binding protein; GPT, Glutamate pyruvate transaminase; NLP, Nodule inception like protein; NIA, Nitrate reductase; gdh, Glutamate dehydrogenase; HO, Heme oxygenase.

During green revolution years, usage of N fertilizers have been an effective way to increase crop performance and productivity. Therefore, optimizing N-metabolism through N-supplementation to saline soils may become as an effective practice for sustainable crop production facilitated by N-assimilation and cellular ion homeostasis (Hessini, 2022; Yin et al., 2022). Additionally, N is interrelated with phytohormones, and play a critical role in the modulation of plant responses to develop salt stress resilience (Ali et al., 2021; Chen et al., 2021). Further, there has been the possibility for significant improvements in nitrogen use efficiency (NUE) through genetic engineering approaches, which have made use of diversified germplasm responses to N supply, to sustain or improve their productivity while minimizing the use of N fertilizers.

The present review is an attempt to elucidate (a) a brief overview of N-sensing, transportation and assimilation in plants, (b) regulation of N metabolism and NUE under salt stress (c) N-supplementation mediated adaptive responses in plants under salt stress. Furthermore, the existing but not much explored crosstalk between N and phytohormones have been discussed to gain a better understanding of plant adaptive responses to salt stress. In addition, this also shades a small beam of light on manipulation of N dynamics through genetic engineering for sustainable agriculture.

## Nitrogen sensing, transportation and assimilation in plants

**Figure 2:**
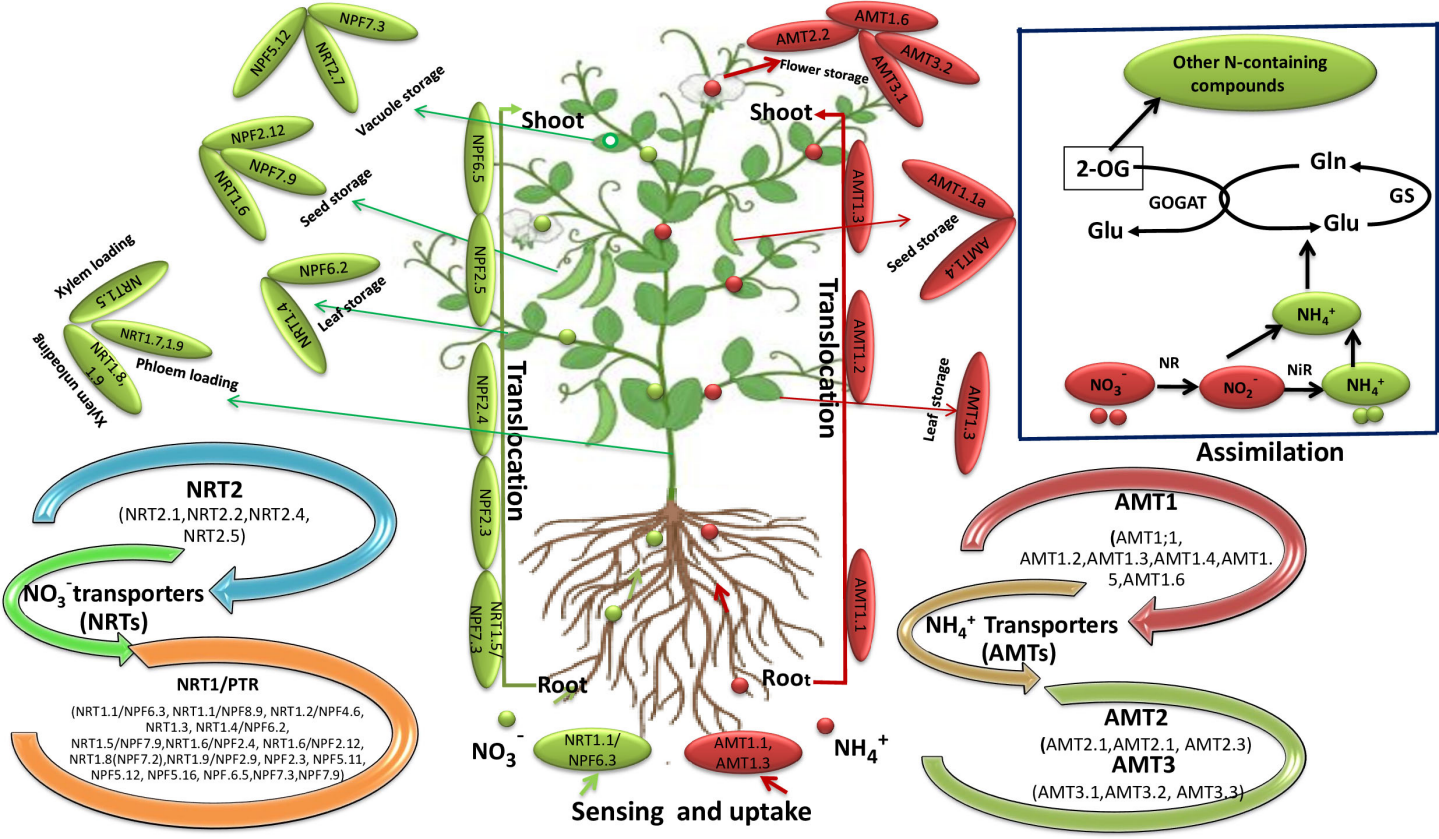
Schematic diagram showing the routes of NO_3_¯ and NH_4_^+^ sensing, transportation and accumulation in plants. The green dots and arrows denote the transport of NO_3_¯ and the red dots and arrows indicates the transport of NH_4_^+^ ions in plants. In the uptake process, NO_3_¯ and NH_4_^+^ enter the plant roots *via* NRTs and AMTs transporters respectively. For example, NRT1.1/NPF6.3/CHL1, AMT1.1 and AMT1.3 involved in the sensing of NO_3_¯ and NH_4_^+^ respectively. These transporter are localized in the plasma membrane of root cells. After uptake, NO_3_¯ transported *via* NRT1.5/NPF7.3, NPF2.3, NPF2.4, NPF2.5 and NPF6.5 and NH_4_^+^ *via* AMT1.1, AMT1.2 and AMT1.3 to the shoot through xylem. NRT1.5 is involved in NO_3_¯ loading in the xylem, while as NRT1.8, NRT1.9 are involved in NO_3_¯ unloading from the xylem. However, NRT1.7, NRT1.9 transport NO_3_¯ into the phloem. Furthermore, NRT1.4/NPF6.2 and AMT1.3 mediates NO_3_¯ and NH_4_^+^ transport to the leaf/petiole. NO_3_¯ accumulation within the leaf vacuole is mediated by NPF7.3, NPF5.12, and NRT2.7. AMT1.1, AMT1.2 and AMT1.3 mediates NH_4_^+^ transoction from root to shoot. NRT1.6/NPF2.12, NPF7.9 and AMT1.1a, AMT1.4 is involved in the transportation of NO_3_¯ and NH_4_^+^ respectively in the seed where they accumulated in the vacuoles. NRT2.7 is a tonoplast transporter of embryo which regulates NO_3_¯accumulation within seed vacuole. AMTs, ammonium transporters; Gla, glutamine; Glu, glutamate; GOGAT, glutamate synthase; GS, glutamine synthetase; NH_4_^+^, ammonium; NiR, nitrite reductase; NO_3_¯, nitrate; NR, nitrate reductase; NRTs, nitrate transporters; 2-OG, 2-Oxoglutarate.

Remarkable progress has been made in understanding the basic mechanisms of how plants sense and adapt to N circumstances. Nitrogen uptake and transportation occurs within and between the plant cells, requiring trans-membrane transporters to facilitate such response. NO_3_^-^ and NH_4_^+^ are the most prevalent forms of inorganic N available in the soil, and a vast number of NO_3_^-^ (NRTs) and NH_4_^+^ transporter families (AMTs) effectively support their absorption and allocation throughout the plant cells and tissues (**Figure 2**). It is known that four families of transporters- NPF family [Nitrate Transporter 1 (NRT1)/Peptide Transporter (PTR) Family], NRT2 family [Nitrate Transporter 2], CLC family [Chloride Channel Family], and SLAC1/SLAH family [Slow Anion Associated Channel Homologs] - contribute to NO_3_^-^ uptake and transport in plants (Xia et al., 2015; Wei et al., 2021; Souza et al., 2022). However, the supply of NO_3_^-^ and the level of plant N nutrition tightly control the functioning of these transporters. For instance, the dual-affinity of NO_3_^-^ transporter like AtNRT1.1 (AtNPF6.3) and the low-affinity transporter-like AtNR1.2 (AtNPF4.6/CHL1) in *Arabidopsis thaliana* (Arabidopsis; Huang et al., 1999; Liu and Tsay, 2003) were reported to be influenced under high NO_3_^-^ conditions. Similarly, the dual-affinity behaviour of NO_3_^-^ transporter, MtNRT1.3 in *Medicago truncatula* (barrel medick; Morere-Le Paven et al., 2011), and the low-affinity NO_3_^-^ transporter ZmNPF7.9 (NRT1.5) in maize (Wei et al., 2021) and OsNPF8.9 (OsNRT1.1, Os3g13274, or AF140606), the OsNRT1.1 allele of OsNPF6.5 (OsNRT1.1B), OsNPF2.4 (OsNRT1.6), and a possible 6-transmembrane NO_3_^-^ transporter OsNRT1.1b (AK066920) in rice (Xia et al., 2015; Fan et al., 2016; Li et al., 2022a) all were shown to be implicated in the uptake of NO_3_^-^. Contrarily, NRT2 family plays an imperative role in NO_3_^-^ acquisition under N deficient conditions (Zou et al., 2020; Li et al., 2022b; Du et al., 2022). For instance, NO_3_^-^ transporter-like NRT2.1, NRT2.2, NRT2.4, and NRT2.5 require a partner protein, NAR2.1 (Kiba et al., 2012; Lezhneva et al., 2014; Zou et al., 2020) in Arabidopsis to acclimatize under such conditions. In addition, other transporters such as CmNRT2.1 in *Chrysanthemum indicum* (Chrysanthemum; Gu et al., 2016), TaNRT2.1-6B in wheat (Li et al., 2022a), BnaZSNRT2s in *Brassica napus* (Canola; Du et al., 2022) and OsNRT2.1, OsNRT2.2, OsNRT2.4 and their partner protein OsNAR2.1 in rice (Naz et al., 2019; Souza et al., 2022) also function as NO_3_^-^ influx transporters (**Figure 2**). Among all NO_3_^–^ transporters, the dual affinity NO_3_^–^ transporter NRT1.1 (AtNPF6.3) serve as a multifunctional protein with a critical role in both NO_3_^–^ acquisition and sensing. Hence, this transporter represents critical step towards understanding the molecular basis of NO_3_^–^ use in plants (Tsay et al., 1993).

The transport and distribution of NO_3_^-^ from root to shoot occurs *via* the xylary tissues (**Figure 2**). Long-distance root to shoot transport in Arabidopsis is mediated by the low-affinity NO_3_^–^ transporter NPF7.3 (AtNRT1.5), which is essential for NO_3_^–^ loading from the root cytoplasm of pericycle cells into the xylem vessel (Sena Falero, 2022). AtNPF7.2 (NRT1.8) and AtNPF2.9 (NRT1.9), negative regulators of root to shoot NO_3_^–^ transport, have promising roles in xylem unloading (Wang and Tsay, 2011). Moreover, NO_3_^-^ is locally stored in vacuoles or assimilated in the cytoplasm of the leaves. According to recent evidences, tonoplast-localized NO_3_- transporters (AtNPF5.11, AtNPF5.12, and AtNPF5.16) could retrieve NO_3_^-^ from vacuoles and transport it to the cytosol, acting as star players in the regulation of NO_3_^-^ allocation between roots and shoots (He et al., 2017). According to de Los Rios et al. (2021), NPF6.2 (NRT1.4), a low-affinity NO_3_^-^transporter, plays a key role in the regulation of leaf NO_3_^-^ homeostasis. TaNRT1.4/AtNPF6.2 constitutively expressed in the midribs and petiole of wheat leaves indicates the role of AtNRT1.4/AtNPF6.2 in the regulation of NO_3_- homeostasis in leaves (Chiu et al., 2004). Moreover, AtNRT1.6/AtNPF2.12 is involved in NO_3_^-^ delivery from maternal tissue to early developing embryos, which influences seed set and NO_3_^-^ acquisition (Almagro et al., 2008). AtNPF5.5, is activated in embryos and its knockout affects N import and accumulation, but apparently leads to unaffected seed development (Léran et al., 2015). AtNRT2.7 is a tonoplast transporter of embryo, up regulated in reproductive organs, which regulate NO_3_- accumulation in the seed vacuole, thus inducing seed germination (Chopin et al., 2007).

OsNPF2.4 and OsNPF7.9 play critical roles in rice, allow the roots to acquire low-affinity NO_3_^-^ by the root, translocation from the root to the shoot, and remobilization from old leaf (source) to young leaf and root (sink organs; Wei et al., 2018; Guan et al., 2022). OsNPF7.3 (OsPTR6) transporter, which is located on the vacuolar membrane, may transport the tripeptide Glycine-Glycine-Leucine (Gly-Gly-Leu) and the dipeptide Glycine-Histidine (Gly-His) (Ouyang et al., 2010). By increasing N absorption and GS activity, it increases rice growth, but when NH_4_^+^ levels are high, it decreases NUE (Ouyang et al., 2010). Additionally, ZmNPF7.9 in maize plays a role in transporting NO_3_^–^ from maternal tissues to the developing endosperm, which could provide valuable knowledge for maize genetic improvement (Wei et al., 2021). Furthermore, the low affinity transporter, VvNPF6.5 modulates NO_3_- uptake and translocation from roots to shoot in *Vitis vinifera* (grapevine) and is implicated in primary NO_3_- response (He et al., 2020).

Under salt stress, NO_3_- dependent transport systems facilitate the uptake and loading of sodium (Na^+^) ion into the xylem, and may represent a key pathway for the accumulation of Na^+^ in Arabidopsis shoots (Álvarez-Aragón and Rodríguez-Navarro, 2017). AtNPF2.4 was much less permeable to NO_3_^−^ and catalysed passive Cl^−^ efflux out of cells. *atnpf2.4* knockdown mutants showed decreased shoot Cl^–^ accumulation, while overexpression of AtNPF2.4 increased shoot Cl^–^ accumulation, suggesting that AtNPF2.4 might function to load Cl^–^ into the xylem of Arabidopsis roots during salt stress (Li et al., 2016). Another permeable to Cl^-^ transporter AtNPF2.5, the closest homolog to AtNPF2.4, has also been shown to serve as a pathway for Cl^-^ efflux from the root, which contributes to the deprivation of Cl^-^ from Arabidopsis shoots under salt stress (Li et al., 2017). Furthermore, under salt stress, AtNPF2.3 promotes NO_3_^-^ translocation from roots to shoots (Taochy et al., 2015). CLCs transporters are also essential for the absorption and allocate Cl^-^ and NO_3_^–^ (Wei et al., 2019; Liu et al., 2020). Rice genome contains five CLC genes, but their underlying mechanisms in NO_3_^–^ translocation have not yet been identified (Diédhiou and Golldack, 2006). In contrast, the tonoplast-localized OsCLC1 and OsCLC2 proteins work to compartmentalize Cl^-^ ions into the vacuole to prevent Cl^–^ induced toxicity under situations of salt stress, and over-expression of OsCLC1 promotes salt tolerance, resulting in higher grain yield in rice (Nakamura et al., 2006; Diédhiou and Golldack, 2006). At the cellular and molecular research, more in-depth research is required to understand the function of various NO_3_^–^ related transporters in response to salt stress in various crop species, which will help to improve agricultural efficiency in the future.

NH_4_^+^ transport across membranes is facilitated by proteins from the NH_4_^+^ transporter/methyl ammonium permeases (AMT/MEP) family in a diverse array of crop species (Giehl et al., 2017; Wu et al., 2019; Bindel and Neuhäuser, 2021). AMT1;4 is primarily restricted to pollen, although four AMT family homologs - AMT1;1, AMT1;2, AMT1;3, and AMT1;5, and one MEP subfamily homolog - AMT2;1 - are expressed in roots of Arabidopsis (Bindel and Neuhäuser, 2021). The primary transporters for high-affinity NH_4_^+^ absorption into Arabidopsis are the root-expressed AMT1-type proteins AMT1;1, AMT1;2, AMT1;3, and AMT1;5 (Giehl et al., 2017; Sasaki and Kojima, 2018; Wu et al., 2019). About two-thirds of the high-affinity NH_4_^+^ absorption capability in roots is accounted for by two of these transporters, AMT1;1 and AMT1;3, which are mostly localized in rhizodermal and cortical cells, including root hairs (Sasaki and Kojima, 2018). It follows that AMT1;2 may mediate the uptake of NH_4_^+^ entering the root *via* the apoplastic transport channel (Giehl et al., 2017; Sasaki and Kojima, 2018). OsAMT1;1 is found in the root stele, epidermis, and mesophyll cells of rice, and knockdown of this gene significantly reduces the mobilization of NH_4_^+^ from the root to the shoot, indicating that OsAMT1;1 is associated with NH_4_^+^ translocation from the root to the shoot (Li et al., 2016). High affinity AMTRs are also highly expressed in the leaf, including NtAMT1.3 in *Nicotiana tabacum* (tobacco; Fan et al., 2017), ZmAMT1.1a and ZmAMT1.3 in maize (Gu et al., 2013), and GhAMT1.3 in *Gossypium hirsutum* (cotton; Sun et al., 2019). In Chinese poplar floral organs, PtrAMT1;5 is stamen-specific, whereas PtrAMT1;6 is female flower-specific (Couturier et al., 2007). In *Sorghum bicolor* (sorghum) floral organs, SbAMT1;1, SbAMT1;2, ​​SbAMT2;1, SbAMT3;1, and SbAMT3;3 are identified in the pistil and stamen, while SbAMT2;2 and SbAMT3;2 are only found in the pistil (Koegel et al., 2013). LjAMT1;1-1;3 expression has also been found in *Lotus japonicus* flowers ((Birdsfoot trefoil; D`Apuzzo et al., 2004). ZmAMT1.1a transcripts have been found in seeds (Zhao et al., 2018). These AMTs might aid in delivering NH_4_^+^ nutrients to the reproductive organs. The exploration of NH_4_^+^ nutrition in plants is still in its early stages and is a largely uncharted area of study.

Ammonium transporter PsAMT1.2 mediates NH_4_^+^ uptake and metabolism under salt stress in Chinese poplar (Li et al., 2021). *PsAMT1.2*-overexpressing transgenic plants with better growth had relatively high ratio of K^+^/Na^+^ compared with the wild type (WT) under salt stress. Similarly, overexpressing the *PutAMT1;1* gene (from *Puccinellia tenuiflora*, forage grass) in Arabidopsis greatly increases its ability to tolerate salt stress during the early stage of root development following seed germination and improved root to shoot NH_4_^+^ mobilization under salt stress (Bu et al., 2019). Zhu et al. (2020) demonstrated that overexpression of *BcAMT1.2* (from *Brassica juncea*, mustard) in Arabidopsis increased the expression levels of N assimilation genes, indicating that an increase in *BcAMT1.2* mRNA abundance could also affect NH_4_^+^ assimilation directly or indirectly.

Organic compounds in the soil can also aid plant N nutrition (Geisseler et al., 2022). Amino acids account for the majority of low molecular weight dissolved organic N in soil (Tsuzuki et al., 2022). Evidence suggests that Arabidopsis has a single target of rapamycin (TOR) kinase gene (*AtTORC*) which is known to be involved in the maintenance of N metabolism including accumulation of amino acids thereby, influencing plant growth and metabolism (Caldana et al., 2013; Dobrenel et al., 2016). It has been revealed that target of TOR is repressed in N-deprived seedlings, however, resupply of N sources such as NO_3_-, NH_4_^+^, amino acids promptly reactivates TOR kinase (Liu et al., 2018). Further, it has been speculated that a number of amino acid permeases (AAP) family, including AAP1, AAP2, AAP3, AAP6, AAP7, AAP8, and AAP16, have a variety of roles in the transport of amino acids (Zhao et al., 2012; Cheng et al., 2016; Wang et al., 2022; Pereira et al., 2022). The families of cationic amino acid transporters (CAT), oligopeptide transporters (OPT), lysine-histidine like transporters (LHT), proline transporters (ProT), and aromatic and neutral amino acid transporters (ANT) have also been identified as having the potential to transport amino acids (Feng et al., 2018; Lin et al., 2019; Guo et al., 2020; Kurt and Filiz, 2022). Only a small subset of these transporters, though, has been essentially described for their function in transporting amino acids. For example, LHT1 aids in the efficient transport of proline and γ- amino butyric acid (GABA) during vegetative and reproductive growth, whereas ProT1 and ProT3 effectively deliver amino acids from vegetative to reproductive organs for grain yield, nutritional quality, and functioning (Lin et al., 2019; Guo et al., 2020).

The coordinated regulation of NO_3_^-^ transporter *McNRT1* and the amino acid transporters *McAAT1* and *McAAT2* from *Mesembryanthemum crystallinum* (crystalline ice plant) mediates the uptake and distribution of nitrogenous compounds and amino acids under conditions of high salt in plants (Popova et al., 2003). Over-expression of *AhProT* (a proline transporter from *Atriplex hortensis*, mountain spinach) had increased proline content in root tips and showed salt tolerance in Arabidopsis (Shen et al., 2002). It is noted that, higher expression level of *ProT1* in Arabidopsis, implying that *ProT1* plays an important role in N distribution during salt stress (Rentsch et al., 1996).

In plants, GS and the GOGAT account for more than 95% of NH_4_^+^ assimilation (Liu et al., 2022). NH_4_^+^ can also be assimilated by glutamate dehydrogenase (GDH), which converts α-oxoglutarate to glutamate. These metabolic intermediates function as important signalling molecules or as significant amino donors for the formation of other amino acids and N-containing compounds, supporting plant growth and development as well as plant responses to stress conditions (Zhi et al., 2020).

## Regulation of nitrogen metabolism and nitrogen use efficiency under salt stress

Regulation of N-metabolism is critical for salt tolerance, and the interaction between salt stress and N is a complex network that influences plant function (Yu et al., 2016; Tian et al., 2022; **Table 1**). Researchers have reported various physiological and molecular processes using a variety of tools ranging from agronomic to genetic approaches for developing salt tolerant plants (Xu et al., 2012; Reddy et al., 2017; Jha et al., 2019). It is known that cultivars that can sustain high NR and GS/GOGAT activities are more resistant to salt stress (Teh et al., 2016; de la Torre-González et al., 2020). On the other hand, species that can retain a higher NO_3_^−^ influx are more resilient to salt stress (Liu et al., 2022). Adequate N supply adjusts and rectifies nutritional disparities in salt-stressed plants. For instance, NO_3_^-^ fed rice and canola plants grew faster and had higher salt toxicity than NH_4_^+^-supplied plants, as evidenced by restricted allocation of Na^+^ in leaf apoplasts (Gao et al., 2016). In contrast, NH_4_^+^ fertilization significantly ameliorated the salt-triggered growth inhibition in sorghum (Coelho et al., 2020). Thus, the effect of N forms on salt resilience varies depending on the plant species. Thus, understanding N metabolism in response to salt stress may be critical for salt tolerance research and will be an interesting research topic for salt stress physiology in the future. Genes involved in N uptake and metabolic processes also play key roles in plant resilience to salt stress. Expression of NO_3_- transporters were down-regulated, while the expression of NH_4_^+^ transporters were up-regulated in roots of salt-stressed- Chinese poplar (Zhang et al., 2014a). Similarly, genes associated with NO_3_- uptake, reduction and N metabolism were down-regulated whereas, NH_4_^+^ transporter genes (*AMT1.2* and *AMT1.6*) were up-regulated in Chinese poplar in response to salt stress (Meng et al., 2016). Furthermore, salt stress up regulated the expression of *OsNRT1.2* and *OsAMT2.1*, while, down-regulated the expression of *OsNRT2.1*, in old leaves of salt-stressed rice (Wang et al., 2012a). Salt stress reduced the expression of *NRT1.1* and *NRT1.2*, which was associated with the reduced NO_3_- content and expression level of *AMT1.2, GS1* and *NR* gene in two cultivars of tomato (Abouelsaad et al., 2016). The genes (*OsGS1.2*, *OsGS2*, *OsNR1*, and *OsFd-GOGAT*) involved in NH_4_^+^ assimilation were down-regulated in rice seedlings due to reduced NO_3_- uptake, resulting in the inhibition of N-assimilation under salt stress (Wang et al., 2012a). The combined action of *NRT1.5/NPF7.3* and *NRT1.8/NPF7.2* regulates the partition of NO_3_- from root to shoot. In response to salt stress, expression of *NRT1.8/NPF7.2* were up-regulated while expression of *NRT1.5/NPF7.3* were repressed in roots which mediates reallocation of NO_3_- back to the roots, and limits NO_3_- translocation to the shoot under stress conditions (Li et al., 2010; Zhang et al., 2014b).

For the world’s sustainable food production, there is an urgent need to improve NUE in agricultural farming systems. Improving NUE under salt stress is critical for plants, as they are increasingly confronted with two major environmental constraints: excessive N fertilization and soil salinization. Several studies have been conducted to assess NUE under saline conditions (Song et al., 2019; Guo et al., 2021; Phan et al., 2022; **Table 1**). It has been proposed that NUE can be determined by agronomic NUE (aNUE), physiological NUE (pNUE), internal NUE (iNUE) and N recovery efficiency (NRE) which gets affected by salt stress. Nitrogen fertilization enhance NUE in rice plants under salt stress as application of low N (0.36 mM) under high salt stress (113 mM) gave better aNUE and agNUE than a high N (2.86 mM) rate under moderate (56 mM) salt stress (Phan et al, 2022). Moreover, salt treatment reduces photosynthetic-NUE in *Capsicum annuum* (chilli pepper; Huez-Lópeze et al., 2011), mustard (Iqbal et al., 2015; Iqbal et al., 2017), and cucumber (Ma et al., 2019). Furthermore, NUE was significantly reduced in the salt-sensitive genotype of *Avena sativa* (oat) compared to tolerant genotype (Song et al., 2019). Recently, Guo et al. (2021) revealed that salt stress significantly reduced N metabolism, NUE, and grain yield in rice seedlings. Effective crop management practices and smart decision-making are required to ensure high crop yields under salt stress conditions and meet increasing global food security. Cultivation of salt-tolerant cultivars is an excellent strategy to increase NUE. Many researchers have found a significant link between NUE and salt resilience of cereal crops. The salt-tolerant genotype of wheat had a higher NUE than the salt-sensitive genotype (Borzouei et al., 2014). High salt stress resistance in maize was attributed to higher NUE attributes such as pNUE, NYE (nitrogen yield efficiency) and NHI (nitrogen harvest index) (Javed et al., 2021). According to Bonnave and Bertin (2018), higher NUE is also responsible for conferring salt resilience in rice. It has been revealed that salt-tolerant wheat lines retained higher yield and yield-related attributes, as well as the maximum NUE indexes, compared to salt-sensitive wheat lines, which could be accountable to the greater N uptake proficiency in the tolerant genotype (Lamichhane et al., 2021). Nitrogen application at the proper dose and timings may also minimize N losses and increase efficiency. It has been reported that moderately low N (2.0 mM) supplementation can help to reduce salt (50 mM, 100 mM)-triggered damage in *Lolium multiflorum* (annual ryegrass) and maximize NUE (Shao et al., 2020). Root stock grafting improves NUE of *Citrullus lanatus* (watermelon) by increasing nutrient uptake and activating NO_3_- and NO_2_- reductase genes (Nawaz et al., 2017). Furthermore, physiological traits that regulates elemental control and the synergy of N partitioning to photosynthesis and remobilized N to sink organs can also be useful for generating high NUE cultivars. Improving N transition to sink organs such as leaves or grains can optimize the NHI and thus the overall NUE (Phan et al., 2022). Salt-stress induced responses also include the accumulation of compatible solutes as well as changes in ion transport (such as uptake, extrusion and sequestration of ions), which may potentially result in the maintenance of redox homeostasis and detoxification, increased NUE and survival under salt stress.

Nitrogen use efficiency is a complex trait controlled by multiple genes. For instance, alanine aminotransferase (AlaAT) and aspartate aminotransferase (AspAT) are involved in the synthesis and degradation of alanine (Ala) and Asp respectively. The increased NUtE under salt stress was attributed to the overexpression of AlaAT and AspAT genes in canola and rice (Beatty et al., 2009; Decui et al., 2020). Accordingly, the activity of AlaAt and AspAT were shown to be increased in *Morus alba* (white mulberry) and *Jatropha curcas* (jatropha) under salt stress conditions (Gao et al., 2013; Ullah et al., 2019). The increased activity of AlaAT and AspAT enzymes may be attributed to high glutamate demands, which maintains the C: N ratio under salt stress conditions, thereby improving NUE (Naliwajski and Skłodowska, 2018). On the contrary, the activity of AlaAT and AspAt were found to be suppressed in wheat and cucumber under salt stress condition (Ahanger and Agarwal, 2017; Li et al., 2019).

Thus, improving NUE in crops is an important goal in agricultural research and our future food production capabilities. Understanding the physiological and molecular mechanisms regulating plant N uptake, assimilation, and redistribution within the cell is critical for enhancing NUE and achieving maximum crop growth, ensuring a better return on investment, and mitigating the adverse effects of salt stress.

## Nitrogen supplementation mediated salt stress tolerance in plants

Nitrogen has been widely reported to be a multifunctional and the most regulating nutrient element for crop production, influencing the primary production of the agricultural system (Melino et al., 2022). The constructive regulatory roles of N-supply in plants to develop salt tolerance have been reported (**Figure 1**, **Table 2**). For instance, supplementation with 50 and 100 mg N kg^− 1^ soil alleviates the deleterious effects of salt stress by activating the antioxidant system and increasing the accumulation of osmolytes and secondary metabolites in wheat plants (Ahanger et al., 2019). Similarly, supplementation of 2.5 mmol L^−1^ N to two cotton genotypes with varying salt stress sensitivity was reported to augment the antioxidant system, accumulation of compatible solutes and ameliorate salt-induced toxicity (Sikder et al., 2020). This revealed the positive role of N by maintaining relative water content (RWC) and protecting the photosynthetic apparatus, especially in the salt-sensitive genotype. Nitrogen fertilizer (105 and 210 kg ha^−1^) applied to salt-stressed wheat plants was reported to have a positive effect on germination rates and seedling characteristics (Ibrahim et al., 2016). In another study, N (86 and 210 kg ha^−1^) significantly decreased the damaging impacts of salt stress and improved germination percentage in wheat seedlings (Ibrahim et al., 2016). In hybrid corn (AG 1051), N application (80 and 160 kg ha^-1^) can ameliorate the deleterious effects of salt stress and enhanced photosynthetic characteristics (Sousa et al., 2021). Furthermore, N application at moderately low levels (2.0 mM) could help in promoting plant growth during salt stress in rye grass by alleviating salt stress induced oxidative damage and reconstitution of N metabolism (Shao et al., 2020). Apart from ensuring physiological homeostasis, N supplementation also affects crop yield under salt stress. A significant improvement in wheat yield traits by application of N fertilizer during salt stress (Ibrahim et al., 2018). Supplementation of N at 200 mg N pot^−1^ ameliorates the detrimental effects of salt stress, which increased plant growth and yield through sustaining the integrity of the photosynthesis and chlorophyll fluorescence processes of oat plants (Song et al., 2019). NO_3_^-^ supplemented wheat and maize plants exhibited greater tolerance and performance as compared to the NH_4_^+^ fed plants under salt stress (Lewis et al., 1989). Additionally, NO_3_- fed *Nerium oleander* (oleander) grows faster with enhanced tolerance to salt stress than NH_4_^+^ fed plants. The higher salt tolerance of NO_3_^-^ fed plants was speculated to be due to decreased transport and accumulation of Na^+^ and Cl^-^ in the shoots, however, accumulation of Na^+^ and Cl^-^ in shoots in NH_4_^+^ fed plants may result in reduced plant growth. Furthermore, enhanced tolerance to salt stress has been observed in Chinese poplar exposed to NO_3_^-^ as compared to NH_4_^+^ as a source of N (Meng et al., 2016), possibly by influencing N uptake, metabolism and accumulation (amino acids, proteins and total N). On the contrary, Hessini et al. (2019) have revealed that NH_4_^+^ favoured the growth of maize plant under salt stress more than NO_3_^-^ by inducing the accumulation of inorganic salts and thereby improving the plant’s capacity to acclimatize to osmotic stress. Shaviv et al. (1990) reported that mixed NH_4_^+^- NO_3_^-^ nutrition effectively mitigated the negative effects of salt stress in wheat, resulting in significantly higher dry matter, grain and protein yields as compared to NO_3_^-^ alone, which may be beneficial to growers desiring ways to maximize grain yield under salt stress conditions. Likewise, Flores et al. (2001) reported that combined NO_3_^-^/NH_4_^+^ regime increase the rate of N assimilation, as well as the levels of Fe and chlorophyll in tomato plants, thereby mitigating salt injury. More research is required to understand the mechanism that regulates N responses. Despite the fact that omics approaches to understanding N-mediated salt stress acclimation responses have not been widely used, there are some reports indicating the interplay of N in plants during salt stress. For instance, transcriptomic analysis of cotton, soybean, alfalfa under salt stress revealed a number of differentially expressed genes (DEGs) related to N absorption (Postnikova et al., 2013; Bahieldin et al., 2015). Overexpression of *Fd-NiR* (ferredoxin-dependent assimilatory nitrite reductase) and *GAD1* (glutamic acid decarboxylase) genes linked to N assimilation and glutamine/glutamine family of amino acids was significantly up-regulated in leaves of mulberry seedlings under salt stress, suggesting that these genes may be important mechanisms for mulberry to acclimatize to salt stress (Zhang et al., 2020a). Furthermore, omics (UPLC-MS and RNA-seq) analysis revealed that N metabolism related genes, encoding xanthin dehydrogenase (XDH), AspAT, AlaTA and allantoinase (ALN), and two hormones (melatonin and (S)-2-aminobutyric acid) in *Beta vulgaris* (sugar beet) were activated under salt stress, which might be attributed to the adaptive response of sugar beet to salt stress (Liu et al., 2020). These studies provides a valuable agronomic and molecular approaches for improving salt stress resistance in plants (**Table 2**). Therefore, further research into the N mediated regulation of plant transcriptome, proteome as well as metabolome will aid in deciphering the N mediated molecular regulatory networks governing salt stress responses in plants, thereby providing new directions for developing salt stress tolerant plants.

**Table 2:** Representative studies on exogenously- sourced nitrogen in regulating various plant processes for adaptation to salt stress.

Plant	Salinity level	Source and level of N applied		Plant responses	References
Mung bean (*Vigna radiata*)	75 mM	Nitrogen fertilizer	60 kg ha^-1^	Improved water relation, accumulation of nutrients and yield	Kabir et al., 2005
Sorghum (*Sorghum bicolor*)	0.6, 6, 8 dS m^-1^	Urea and ammonium nitrate	137 kg ha^-1^	Increase uptake of N, P, Ca Mg and decreased Na^+^ and Cl^-^ concentration	Esmaili et al., 2008
Cotton (*Gossypium hirsutum*)	7.7, 12.5 dS m^-1^	Urea	135 and 270 kg ha^−1/^	Enhance N uptake and alleviates adverse effects of salinity	Chen et al., 2008
Maize (*Zea mays*)	12.4 dSm^−1^	Urea and Nitrate	100 kg ha^−1^	Improved nitrogen use efficiency	Irshad et al., 2008
*Suaeda physo phora*	75 mmol L^−1^	Nitrate	10 mM L^-1^	Increased root:shoot ratio, effective N uptake in shoots and lateral root development	Jun-Feng et al., 2010
Seep weed (*Suaeda salsa*)	42 g	Urea	4.8 g	Increased the biomass of leaf, shoot and root, regulation of Na^+^, K^+^ and Cl^-^ accumulation	Guan et al., 2011
Wheat	100, 150 and 200 mM	Nitrogen fertilizer	86 and 210 kg ha^−1^	Improved germination percentage, shoot and root length, dry weight, salt tolerance index, and seedling vigor index	Ibrahim et al., 2016
Mustard (*Brassica juncea*)	50 mM	Nitrogen fertilizer	100 mM	Enhanced antioxidant system	Naveed et al., 2019
Wheat (*Triticum aestivum*)	1.4 and 2.5 dS m^−1^	Urea	86 kg ha^-1^	Accumulation of antioxidant enzymes, improved seedling growth	Ibrahim et al., 2019
Oat (*Avena sativa*)	100 mM	Nitrogen fertilizer	50,100, 200 kg ha^−1^	Improved photosynthetic rates, grain yield and other yield components	Song et al., 2019
Wheat	100 mM	Urea	50 and 100 mg kg^− 1^ soil	Increased osmolyte and secondary metabolite accumulation, and redox components in N supplemented plants regulated the ROS metabolism and salt tolerance	Ahanger et al., 2019
Rice (*Oryza sativa*)	2.5-3.5 dS m^-1^	Urea	300 kg ha^-1^	Enhanced antioxidant system and solube sugars content, and improved grain yield	Zhu et al., 2020
Regrass *(Lolium multiflorum)*	50 and 100 mM	Nitrogen fertilizer	2.0 mM	Promoted growth *via* regulating photosynthesis, alleviating ROS-induced damage and maintenance of N metabolism.	Shao et al., 2020
*Gossypium hirsutum* (cotton)	200 mm.L^-1^	Nitrate	2.5 mmol L^−1^	Enhanced the accumulation of osmolytes, such as soluble sugars, soluble proteins, and free amino acids.	Sikder et al., 2020
Hybrid corn (AG 1051),	3.0 dS m^-1^	Nitrogen fertilizer	80 and 160 kg ha^-1^	Increased plant height, leaf area, photosynthesis, transpiration, and conductance	Sousa et al., 2021

## Crosstalk of nitrogen with phytohormones to induce salt stress tolerance

**Figure 3:**
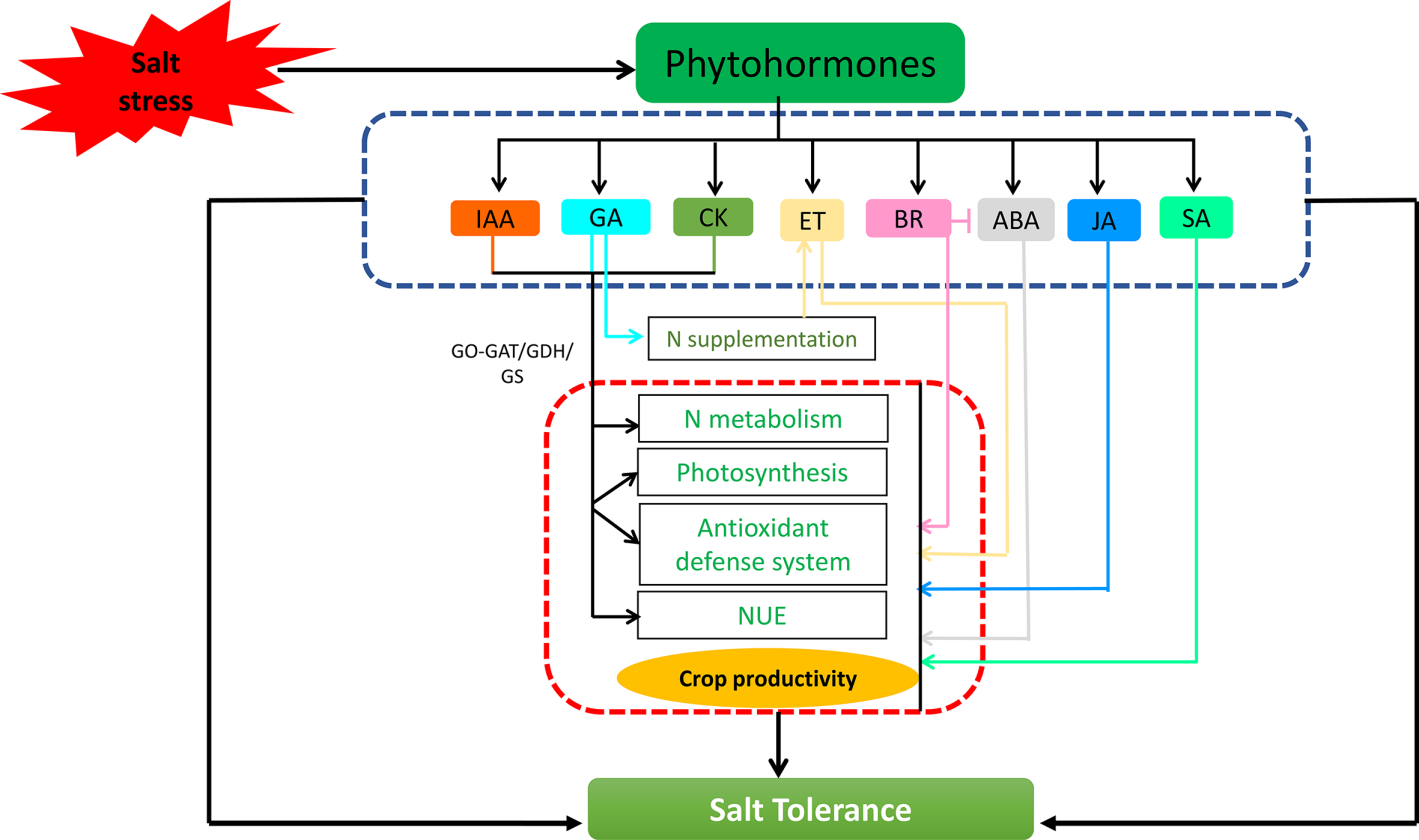
Phytohormones have been found to regulate nitrogen metabolism during salt stress, either in conjugation with nitrogen sources or by interacting with other phytohormones leading to improved physio-biochemical attributes, and promoting plant growth and development under salt stress.

Phytohormones, besides controlling plant growth and development under normal conditions, may also play a critical role in how plants respond to salt stress, which could lead to increased plant growth and productivity (Yu et al., 2020). Both N and phytohormones interact in a complex system with diverse synergistic and antagonistic interactions, resulting in plant responses under normal and salt stress conditions (**Figure 3**). For example, *Vigna radiata* (mungbean) plants sprayed with indole-3-acetic acid (IAA), gibberellic acid (GA) and kinetin (CK) ranging from 0.1**-**10 µM significantly increased the activity of key enzymes of N metabolism (GS, GOGAT and GDH) along with minimizing the effects of salt stress in plants (Chakrabarti and Muhkerji, 2003). Thus, it is possible that there is a complex crosstalk between N, IAA, GA and CK that modulates salt stress-induced mitigation responses in plants that has yet to be comprehended. Application of N (90 and 135 kg N ha^-1^) or GA_3_ (144.3 and 288.7 μM) alone or in combination reversed the inhibitory effect of salt stress in sorghum, making it a useful approach for maintaining cellular homeostasis, photosynthesis, lowering oxidative damage, and improving and managing overall crop productivity under salt stress (Ali et al., 2021). Similarly, application of N (40 mg N kg^-1^) or GA_3_ (10^-5^ M) alone or in combination proved to be a physiological remedy to enhance the resistance against the adverse effects of salt stress in mustard (Siddiqui et al., 2008), thus indicating that a more complex N–GA interaction may be involved in salt stress responses. The crosstalk of N with ethylene is also important in plant cellular metabolism, and it has the capability to deal with salt stress. In mustard, cumulative treatment of ethephon with split doses of N and sulphur (S) mitigated salt-induced damages by improving photosynthetic efficiency, assimilation of N and S, proline content and antioxidant defense system and decreased generation of oxidative stress markers (Jahan et al., 2021). In another study, foliar spraying of ethylene (2-chlorethyl-phosphonic acid) to salt- exposed *Pennisetum typhoides* (pearl millet) seedlings ameliorated salt- induced toxicity mainly by increasing the activity of several enzymes associated with N metabolism as well as improving protein synthesis and proline levels (Eder et al., 1977), which could play a significant role in sustaining crop productivity and nutritional quality. Moreover, ethephon have been found to act synergistically with N to improve photosynthesis in mustard by modulating the pNUE and antioxidant metabolism under salt stress conditions (Iqbal et al., 2017). An interaction between N and ethylene along with proline was also revealed in salt- exposed mustard, where N supplementation significantly increased photosynthetic efficiency mainly by regulating proline and ethylene production (Iqbal et al., 2015). More research is needed to gain a better understanding of N and ethylene-mediated salt stress alleviation. Furthermore, a functional interaction between abscisic acid (ABA) and N in mitigating salt stress resilience has been reported in mustard, where ABA (25 µM) in coordination with N (10 mM) ameliorates salt- inhibited photosynthetic efficiency by increasing the activity of antioxidant enzymes and accumulation of proline, which maintains osmotic balance and reduces oxidative stress (Majid et al., 2021). However, more researches are required to promote the differential roles of N and ABA under salt stress, which could aid in improving plant resilience traits. The cumulative treatment of N supplementation (3 mM) and seed priming with 24- epibrassinolide (24-EBL;100 mM) significantly enhanced salt stress tolerance in soybean plants by increasing antioxidant activity and osmolyte accumulation, resulting in improved photo-protection through maintenance of tissue water content (Soliman et al., 2020). Furthermore, the application of EBL (10^-7^ M and 0.5 × 10^-9^ M) to pigeon pea reduced the adverse effects of salt stress by increasing NO_3_^-^ uptake, which was associated with increased activity of NR along with increased levels of free amino acids and soluble proteins in salt- stressed plant roots, showing the significance of brassinosteroid (BR) synthesis for plants growing under salt stress (Dalio et al., 2013). In another report, co- application of EBL and sodium nitroprusside (SNP) along with N and ABA was more effective than their individual treatments in decreasing salt- induced oxidative stress in mustard (Gupta et al., 2017). The positive effect was accomplished by reducing Na^+^ acquisition, oxidative stress and increasing proline content. Thus, N appears to have an intriguing crosstalk with BRs in modulating salt stress amelioration. Salicylic acid (SA) and N are also implicated in an intriguing crosstalk that regulates plant metabolism and can mitigate the negative impacts of salt stress. For instance, SA and N prevented salt- induced adversities in two mungbean cultivars by improving photosynthetic efficiency, as well as modulating N, S and antioxidant metabolism (Nazar et al., 2011). It has been revealed that treatment of SA (0.5 mM) improved the efficacy of salt- stressed mungbean plants by increasing the activity of N assimilatory enzymes, proline accumulation and defense system, while decreasing the content of Na^+^ and Cl^-^ ions, resulting in increased photosynthesis and growth (Hussain et al., 2021). Furthermore, Yang et al. (2019) explored the role of methyl jasmonate (MeJA) in mitigating salt- induced damages in *Glycyrrhiza uralensis* (Chinese liquorice) mainly by increasing the activity antioxidant defense system and modulating N-metabolism, which in turn regulates the survival of these plants under salt- stress conditions. Thus, N has gained popularity as an essential macro element capable of inducing salt stress-adaptation in plants. It mainly revitalises the reservoir of antioxidants, necessary to detoxify the toxic reactive oxygen species (ROS) produced under salt stress. Given the importance of N as a major nutrient in agricultural systems, and its crosstalk with different phytohormones, it can be further utilised to strengthen the production of more tolerant varieties to salt stress. To date, there is scant data that elucidates the potential crosstalk of N and phytohormones-triggered responses under salt stress. More in-depth and convincing research is needed at the cellular and molecular levels under salt stress, to explore the intrinsic signalling mechanism of N-phytohormone interactions that would provide new insights into the complex signalling pathways, which could be used to increase the yield and stress resilience under today’s changing environment. These findings will benefit not only plant scientists but also breeders and will open up new avenues of research into plant responses to salt stress.

## Manipulation of nitrogen dynamics through genetic engineering

**Figure 4:**
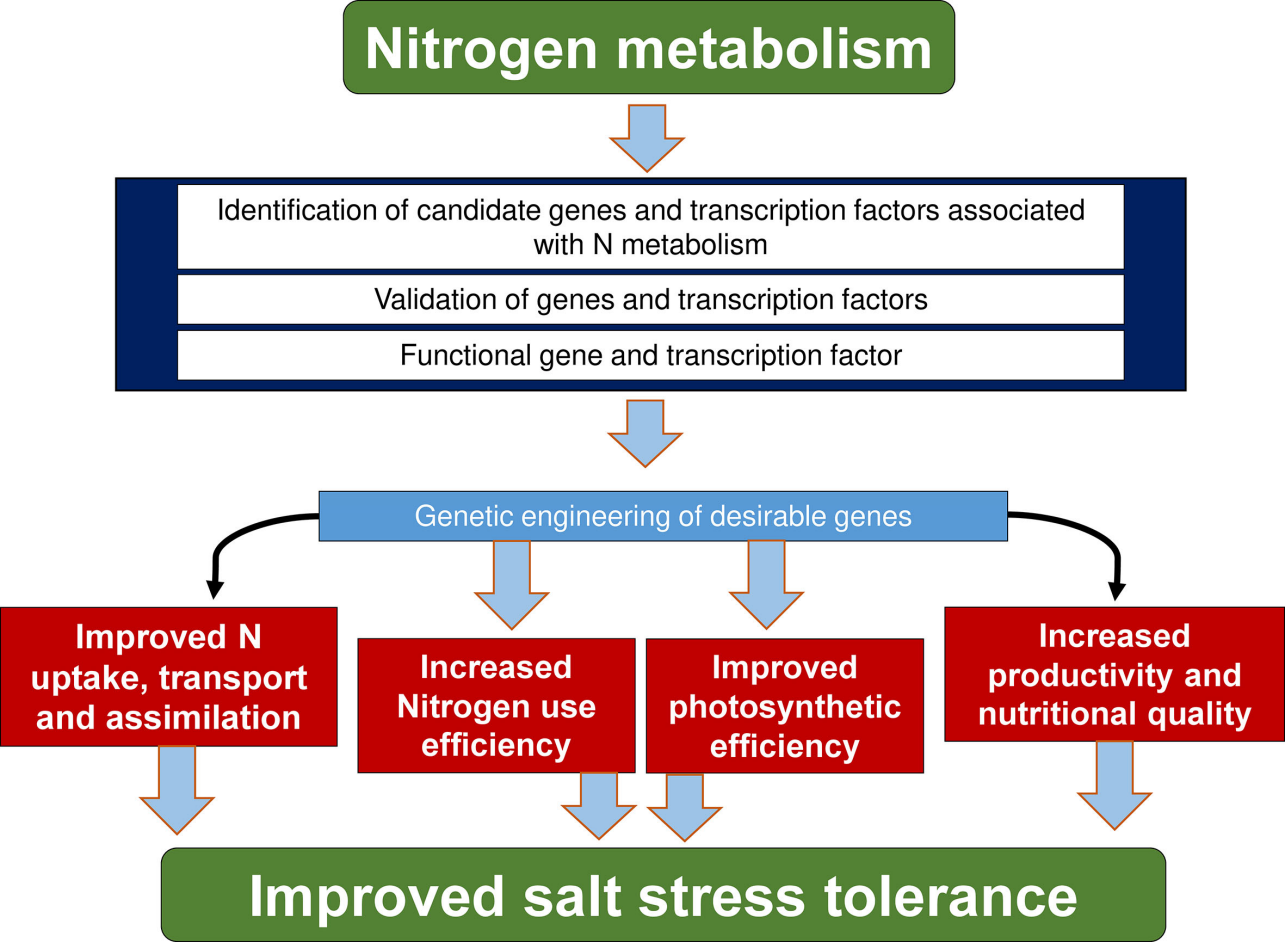
Specific targets for manipulating nitrogen dynamics through genetic engineering. Nitrogen dynamics is mainly associated with higher expression of nitrate and ammonium transporter genes as well as transcription factors, and the overexpression of these genes in transgenic plants leads to increased nitrogen use efficiency, photosynthesis, crop productivity and nutritional quality, nitrate and ammonium uptake and salt-stress resistance.

Engineering metabolic pathways by reinforcement of gene identification associated with N metabolism and modifying their key genes expression have emerged as an important factor for ameliorating constraints from crop nutrition and for improving crop quality. Generating transgenic crops by modulating the expression of genes involved in uptake, transport and distribution of N can help in maintaining N homeostasis and promoting plant growth under normal or stressful conditions (**Figure 4**). For instance, overexpression of *OsAMT1;1* in transgenic rice greatly improved N uptake, translocation capacity, and aided in the maintenance of N homeostasis, resulting in higher NUE, amino acid levels, photosynthetic pigments, and sugars, as well as increased grain yield, particularly under both suboptimal and optimal N fertilizer environments (Ranathunge et al., 2014). Likewise, simultaneous activation of the *OsAMT1;2* and *glutamate synthetase 1 (OsGOGAT1)* genes in rice serves as an efficient breeding strategy to improve plant growth, NUE, and grain yield, particularly under N limitation, by improving both N uptake and utilization (Lee et al., 2020). Overexpression of the high-affinity urea transporter gene, *OsDUR3* (from rice) improves urea acquisition and allocation in transgenic Arabidopsis roots (Wang et al., 2012b). Under N-deficit environments, overexpression of maize *Dof1*, an activator of genes involved in organic acid metabolism improves plant growth and amino acid levels (Gln and Glu), decreases glucose levels, improves N-assimilation, and enhances N-content in Arabidopsis (Yanagisawa et al., 2004). *ZmDof1* overexpression enhances N uptake, assimilation, and N and C levels in transgenic rice, resulting in increased photosynthetic rate and biomass in transgenic rice (Kurai et al., 2011). The high-affinity nitrate transporter gene (*OsNRT2.1*) has been widely used for the production and remodelling of transgenic rice with optimized NO_3_^-^ utilization, which is critical for maintaining crop yield under low N conditions (Katayama et al., 2009). Additionally, under both limited and sufficient N conditions, overexpression of *TaNRT2.1-6B* (dual-affinity NO_3_^-^ transporter) increased N influx, root growth, SPAD value, grain yield, and NUE in wheat, whereas gene silence lines had the inverse effects (Li et al., 2022a). Similarly, in *Hordeum vulgare* (barley), overexpression of the high affinity NO_3_^-^ transporter, *OsNRT2.3b* under the influence of ubiquitin promoter optimized yield, NUE, and regulation of nutrient uptake and homeostasis (Luo et al., 2020).

Under salt stress condition, transgenic rice plant over-expressing *GS2* (chloroplastic glutamine synthetase) had enhanced photorespiration capacity and salt tolerance. This study decoded the role of *GS*2 in maintaining intracellular K^+^ and Na^+^ homeostasis, which has important implications for plant response to salt stress (Hoshida et al., 2000). Transcriptomic studies revealed that genes associated with N metabolism (*NR, NIR)* were substantially up- regulated in seagrass (*Zostera marina*) under salt stress, suggesting this may be an adaptation for NO_3_- absorption, transport and assimilation in a high salt environment (Lv et al., 2018). In addition, the genes (NR) and metabolites (amino acids) related to N metabolism have been found to be strongly regulated by salt stress in tomato (Mellidou et al., 2021). Concurrent overexpression of *OsGS1;1* and *OsGS2* isoforms in rice improved photosynthetic and agronomic performance under salt stress during the reproductive stage (James et al., 2018). Furthermore, most NRTs were found to be overexpressed in *Sophora japonica* (Japanese pagoda tree) under salt stress, allowing the root to maintain high NO_3_-uptake ability (Tian et al., 2022). Moreover, *BjNRT1.3*, *BjNRT1.4* and *BjNRT1.8* were up-regulated under salt (1h, 24h) and osmotic (1h) stress in mustard while the down-regulation of *BjNRT1.1, BjNRT1.5, BjNRT2.1*, *BjAMT1.2* and *BjAMT2* after stress exposure, suggesting their involvement in plant tolerance mechanism to salt stress (Goel and Singh, 2015). Expression of splice variant of *OsNRT2.3* (*OsNRT2.3b*) has been identified for mediating cytosol pH sensing and balancing the uptake of NO_3_- and NH_4_^+^ in response to external perturbations of N supplies (Fan et al., 2016). Surprisingly, the high affinity tomato (*SlNRT2*) gene has been found to be involved in facilitating adaptive responses in plants exposed to salt stress (Akbudak et al., 2022). It has been revealed that salt stress induces the expression levels of both *GLN-1.1* and *GLN-1.2* in Arabidopsis roots probably due to higher demand of GS or other N-metabolism related enzymes required by the plant as an adaptation against higher level of salt stress (Debouba et al., 2013). Recently, Sathee et al. (2021) have shown that rice genotypes differing in reproductive stage salt tolerance have different transcript abundance of *NIA2, GLN2, GLN1.1* and *GLN1.2, Fd-GOGAT* and *NADH-GOGAT* genes; the expression levels was found higher in salt tolerant than salt sensitive cultivar, suggesting its protective role against salt-induced oxidative damage. Ectopic expression of *MdNIA2* from *Malus domestica* (apple) in *Arabidopsis* and apple callus elevated the NUE and increased root hair elongation and formation, resulting in promoted plant growth and salt stress tolerance (Liu et al., 2023). Thus, using transgenic strategies to manipulate the N status of plants has aided in plant growth, increased N content, uptake, and remobilization for combating food insecurity, and improved productivity to foster sustainable agriculture programmes.

In recent times, researchers have uncovered a plethora of transcription factors (TFs) that regulate the transcription of targeted genes involved in absorption, redistribution, and assimilation of N. The majority of these TFs have been used to boost NUE in crop plants. The NAC superfamily is one of the most extensive and critical plant-specific TF families. For instance, NO_3_^−^-inducible NAC TF (TaNAC2-5A) was identified in wheat, binding to the promoter region of genes involved in N transport and assimilation, its overexpression improved root growth, NO_3_^−^ influx rate, grain yield, and thus increased the root’s capacity to accumulate N under N- limited environments (He et al., 2015). Similarly, OsNAP (an apetala3/pistillata-activated NAC-like), a member of the NAC TF family, is regulated by ABA and mediates age-related senescence (Liang et al., 2014). This TF binds to the promoter region of the nutrient transporter and regulates N re-translocation for grain filling in rice leaves. Overexpression of *OsNAP* increased grain N content in rice, its knockdown reduced grain yields with impaired leaf senescence (Liang et al., 2014). Furthermore, salt stress induced the expression of NO_3_^-^ responsive transcript factor, OsNLP2 in rice during early stage of seed germination, which was accompanied by an increase in *OsNR1/2* and NR activity, resulting in improved salt tolerance (Yi et al., 2022). Similarly, another nitrate responsive TF (OsMADS27), positively regulates salt tolerance in rice in a NO_3_^–^ dependent manner by regulating salt-responsive genes, maintaining nutrient homeostasis and modulating the expression of genes related to N uptake and assimilation (Alfatih et al., 2022). OsMYB305, a MYB transcription factor, is mainly expressed in young rice panicles, but it is also expressed in roots when N levels are low. Rice plants overexpressing this TF exhibited increased plant growth, more tiller number and enhanced N uptake and assimilation than WT. The increased NO_3_^-^ uptake in the overexpression lines, associated with the increased expression of N-related genes *(OsNRT2.1, OsNRT2.2, OsNAR2.1*, and *OsNIR2*) in roots suggests that OsMYB305 could be a potential candidate for rice NUE improvement (Wang et al., 2020). Higher NUE of indica than japonica is associated with a natural transformation of OsMYB61, whose expression is restrained by growth regulatory factor 4 (GRF4), a C/N metabolic regulator in rice (Gao et al., 2020). OsMYB61 expression was induced by a low supply of N, and this effect was more pronounced in the indica than in the japonica subspecies. Transcription factors belonging to basic leucine zipper (bZIP) family play an intrinsic part in the manipulation of N dynamics and resilience to abiotic stresses (Yang et al., 2019). Overexpression of TabZIP15 in transgenic wheat plants improves salt stress tolerance and root development (Bi et al., 2021). AtbZIP24 increased salt tolerance by maintaining osmotic balance, amino acid levels (Gln and Glu) and increased growth and development, involving homo- and heterodimerization, or post-transcriptional modification (Yang et al., 2009). Other TFs, rice Nin-Like Protein 1 (OsNLP1) have also been implicated in N utilization (Alfatih et al., 2020). Over-expression of this gene was found to be significant in improving rice yield and NUE under diverse N environments, whereas knockout of the gene with the CRISPR/Cas9 system exerted negative impacts on crop productivity at low as well as under normal N conditions, but no significant change was observed at high N levels. Overexpression of FtWRKY46 derived from Tartary buckwheat in Arabidopsis increased salt tolerance by regulating ROS detoxification and stress-related gene expression (Lv et al., 2020). Reaumuria trigyna derived RtWRKY1 confers tolerance to salt stress in transgenic *Arabidopsis* by regulating plant growth, N-metabolism, osmotic balance, Na^+^/K^+^ homeostasis, and the antioxidant system (Du et al., 2017). Thus, the aforementioned studies opens up new possibilities and fill a gap in our understanding of the dynamics of NUE and crop productivity. More convincing research is needed to support the differential role of genes and TFs encoding NH_4_+ and NO_3_- assimilation enzymes that regulates N metabolism in plants under salt stress.

## Conclusion and future prospective

In conclusion, salt stress poses a serious threat to soil fertility and productivity of agriculture. Correct plant mineral nutrition has often been shown to have a protective role in bestowing salt stress tolerance and in sustaining crop production. Here, we provided convincing evidence for N-mediated salt stress tolerance in plants by up-regulating salt stress tolerance genes. The use of N as fertilizer or soil supplementation with N-containing compounds in agriculture system may emerge as a useful tool for combating salt-induced effects and regulating N metabolism and NUE under salt stress conditions. There are still some gaps in our understanding of crosstalk of N with phytohormones that need to be filled in order to improve salt stress tolerance. Despite significant progress, there is an urgent need to develop more understanding of N-dynamics in the crop improvement against salt stress. The identification and analysis of key genes would be effective in N-mediated salt tolerance, providing a new framework for developing crop varieties with improved NUE to meet the requirements for salt stress tolerance and sustainable agriculture. Furthermore, the advent of genetic engineering technology holds significant promise for crop improvement and stress management.

## Author contributions

Conceptualization, MIRK. Software, FN and FA. Writing- Original draft preparation, FN, FA, HC and PC. Writing – Review and editing, MIRK, FN, MM, SK and MA. All authors contributed to the article and approved the submitted version.

## Glossary

**Table:**

AAPs	amino acid permeases
AMT	ammonium transporter
APX	ascorbate peroxidase
BABA	β-aminobutyric acid
CLC	chloride channel family
DEG	differentially expressing genes
GOGAT	glutamate synthase
GS	glutamine synthetase
G6PHD	glucose-6-phosphate dehydrogenase
LHTs	lysine/histidine transporters
NAC	NAM (no apical meristem), Petunia, ATAF1–2 (*Arabidopsis thaliana* activating factor), and CUC2 (cup-shaped cotyledon, Arabidopsis)
NCC	nitrogen containing compounds
NH_4_^+^	ammonia
NiR	nitrite reductase
NO_2_^-^	nitrite
NO_3_^-^	nitrate
NR	nitrate reductase
NRT2	nitrate transporter 2
NUE	nitrogen use efficiency
NRE	nitrogen recovery efficiency
ProTs	proline transporters, GBTs, glycine betaine transporters
PTR	peptide transporter family
QAC	quaternary ammonium compounds
SLAC1	slow anion channel homologs
